# Survival outcomes after traumatic brain injury during national academic meeting days in Japan

**DOI:** 10.1038/s41598-021-94759-4

**Published:** 2021-07-26

**Authors:** Sanae Hosomi, Tetsuhisa Kitamura, Tomotaka Sobue, Hiroshi Ogura, Takeshi Shimazu

**Affiliations:** 1 Department of Traumatology and Acute Critical Medicine, Osaka University Graduate School of Medicine, 2-15, Yamadaoka, Suita, Osaka 565-0871 Japan; 2 Division of Environmental Medicine and Population Sciences, Department of Social and Environmental Medicine, Osaka University Graduate School of Medicine, 2-15, Yamadaoka, Suita, Japan

**Keywords:** Medical research, Neurology, Risk factors

## Abstract

Surgeons and medical staff attend academic meetings several times a year. However, there is insufficient evidence on the influence of the “meeting effect” on traumatic brain injury (TBI) treatments and outcomes. Using the Japan Trauma Data Bank, we analyzed the data of TBI patients admitted to the hospital from 2004 to 2018 during the national academic meeting days of the Japanese Association for Acute Medicine, the Japanese Society of Intensive Care Medicine, the Japanese Association for the surgery of trauma, the Japan Society of Neurotraumatology and the Japan Neurosurgical Society. The data of these patients were compared with those of TBI patients admitted 1 week before and after the meetings. The primary outcome was in-hospital death. We included 7320 patients in our analyses, with 5139 and 2181 patients admitted during the non-meeting and meeting days, respectively; their in-hospital mortality rates were 15.7% and 14.5%, respectively. No significant differences in in-hospital mortality were found (adjusted odds ratio, 0.93; 95% confidence interval, 0.78–1.11). In addition, there were no significant differences in in-hospital mortality during the meeting and non-meeting days by the type of national meeting. In Japan, it is acceptable for medical professionals involved in TBI treatments to attend national academic meetings without impacting the outcomes of TBI patients.

## Introduction

Traumatic brain injury (TBI) is a major cause of death and permanent disability worldwide^1^. Management of moderate to severe TBI alone and in combination with extracranial lesions usually starts in the emergency room (ER) with initial resuscitation before transfer to the intensive care unit (ICU). A combined medical-surgical approach is employed by multidisciplinary teams consisting of different medical professionals, such as ER physicians, intensivists, neurosurgeons, trauma surgeons, as well as ER, operation room (OP) and ICU nurses, and biomedical equipment technicians^2^. Dedicated trauma teams led by trained trauma surgeons have yielded improved outcomes, particularly among severely injured patients^3,4^. Additionally, implementing dedicated trauma care units and involving trauma-specific physician assistants seem to be beneficial^5^.

In this sense, the composition of hospital staff during national meeting days or off-days is thought to be associated with increased mortality risk for critically ill patients and those requiring emergent surgical interventions^6–9^. Annually, most medical staff attend national academic meetings. While participating in these meetings can positively impact daily medical practice, there might be a decrease in medical staff during the meeting days. Despite the growing evidence supporting the value of specialized teams and ICUs for complex patient populations, such as TBI and trauma patients, the relationship between post-TBI survival and attendance of medical staff in national academic meetings related to trauma or ICU has been insufficiently examined.

The Japanese Trauma Data Bank (JTDB) was launched in 2003 by the Japanese Association for the Surgery of Trauma (JAST; Trauma Surgery Committee) and the Japanese Association for Acute Medicine (JAAM; Committee for Clinical Care Evaluation)^10^. It is similar to trauma databases in North America, Europe, and Oceania. Since 2018, 272 major emergency medical institutions across Japan have been registered in the JTDB database^10^. The service level of the included hospitals was similar to Level I trauma centers in the USA. Using this database, we evaluated differences in in-hospital survival after TBI during national meeting and non-meeting days in Japan. We hypothesized that there would be increased TBI-associated mortality during the national meeting days because many ER physicians and neurosurgeons attend these meetings.

## Materials and methods

### Study design, population, and setting

This retrospective cohort study used data from the JTDB database. We included TBI cases registered in the database from January 2004 to December 2018 and those transported to JTDB-participating hospitals. TBI (using the Abbreviated Injury Scale [AIS] code) was defined as a parenchymal or vascular lesion inside the skull, i.e., in the brainstem, cerebellum, and the cerebrum^11^. Other than isolated TBI, cases with critical injuries in other regions (AIS ≥ 3) were also defined for polytrauma^12^. We excluded patients with a maximum head AIS score of 6 (non-survivable injury) or 9 (unspecified injury), those requiring inter-hospital transport, or those who underwent cardiopulmonary arrest upon hospital arrival^13^. Patients who underwent cardiopulmonary arrest were defined as those with a systolic blood pressure (SBP) of 0 mmHg and/or heart rate of 0 bpm on or before hospital arrival^14^. We also excluded cases with missing data on age, sex, Glasgow Coma Scale (GCS) score on arrival, Injury Severity Score (ISS), or survival outcome.

This study was approved by the ethics committee of Osaka University Graduate School of Medicine (No. 16260). Personal identifiers were removed from the JTDB database beforehand; thus, the need for informed consent was waived.

### Japanese trauma data bank

Data were collected via the Ιnternet from the participating institutions. Data were mainly entered by physicians and medical assistants working at JTDB-registered hospitals after they had attended an AIS-coding course^15^. The JTDB records trauma patients’ data, including age, sex, mechanism of injury, AIS code (version 1998), ISS, vital signs upon hospital arrival, date and time of hospital arrival and discharge, medical treatments (such as interventional radiology, surgical operation, and computed tomography findings), complications, and in-hospital death^15^. The ISS was calculated from the top three AIS scores in nine sites classified using the AIS codes. The data used in this study are the most recent available data from this registry.

### Key group definition

In this study, we focused on five national academic meetings, the JAAM^16^, Japanese Society of Intensive Care Medicine (JSICM)^17^, JAST^18^, Japan Society of Neurotraumatology (JSNT)^19^, and Japan Neurosurgical Society (JNS)^20^. This is because we believe that medical professionals, such as physicians, nurses, and medical engineers belonging to these academic societies, may play important roles in treating severe TBI patients after hospital admission. The JAAM, JSICM, JAST, and JNS have a total of 10,150, 9117, 2208, and 9940 members^21^ and 5439^16^, 2127^17^, 231^18^, and 7903^19^ specialists, respectively. The JNST has 814 members; however, their first examination for selecting specialists commenced in 2021^20^. The national meetings of JAAM, JSICM, and JNS usually last 3 consecutive days every October, March, and September, respectively, while the JAST and JSNT meetings last 2 consecutive days in May and February, respectively. Calendar days at these meetings were obtained for each year during the study period.

For analyses, we used the double-control method based on previous studies^22^, which allows near-perfect temporal symmetry for cases and controls and does not create time imbalance inside each pair to assess outcome differences during the exposure and control periods. In accordance with this method, we identified two groups: the exposure group, which included patients with TBI occurrence on meeting days, and the control group, which included patients with TBI occurrence during the same day of the week, 1 week before and after meetings.

### Main outcome measures

The primary outcome of this investigation was in-hospital death, and the secondary outcomes were remergency department (ED) mortality, time from onset to arrival at the hospital, time from arrival to operation commencement, and duration of hospital stay.

### Statistical analysis

Descriptive data are presented as counts and percentages (categorical variables) or as medians with interquartile ranges (numerical variables). Patients’ characteristics and hospital care among the eligible TBI patients were compared between the exposure and control groups using Student’s t-test and the χ^2^ test for categorical variables. Multivariable analysis with logistic regression models was used to compare differences in mortality outcomes between the two groups; adjusted odds ratios (ORs) and 95% confidence intervals (CIs) were calculated. Clinically significant confounders were carefully selected from previous reports and adjusted for analyses^6–9,23–26^. In the multivariable logistic regression model, we adjusted for the following 12 variables: age groups (age < 18, 18‒64, and ≥ 65 years), sex (male, female), type of injury (blunt, other), mechanism of trauma (traffic accident, fall, and other), transfer system (ambulance, car/helicopter with physician, other), GCS group on arrival (severe: GCS score, 3‒8; moderate: GCS score, 9‒12; mild: GCS score, 13‒15), hypotension (defined as SBP ≤ 90) on ED admission (no, yes), operation indicated for TBI (no, yes), use of anticoagulant or antiplatelet drugs (no, yes), maximum head AIS scores^3–5^, multiple trauma (no, yes), and ISS. Additionally, the adjusted ORs were calculated for each meeting.

Statistical significance was defined as two-sided p-values < 0.05 for patient characteristics or was assessed using the 95% CI for mortality in all statistical analyses. Analyses were performed using STATA version 16 (StataCorp, College Station, TX, USA).

The study was conducted in accordance with the Declaration of Helsinki. This manuscript was written based on the STROBE statement for comprehensive reporting of cohort and cross-sectional studies^27^.

## Results

### Patient characteristics

**Figure 1:**
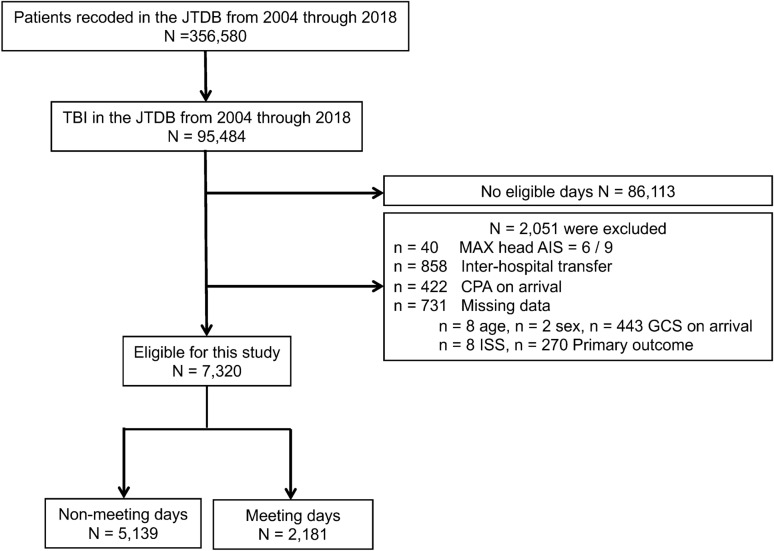
Flow chart of patients included in this study. *JTDB* Japan trauma data bank, *AIS* abbreviated injury scale, *CPA* cardiopulmonary arrest, *GCS* glasgow coma scale.

During the study period, a total of 95,484 cases of TBI were documented (Fig. 1). After excluding victims during the non-eligible days, the data of 7320 participants (2181 in the exposure group and 5139 in the control group) were included for analyses.

The characteristics of patients with TBI events during the academic meeting and non-meeting days are presented in Table 1. Among victims in both groups, the proportion of men was approximately 67%, and the mean age was 66 years. There were no differences between meeting and non-meeting days regarding the mechanism of trauma, transfer system, type of TBI, and proportion of surgeries for TBI. The proportion of hypotension on arrival, low GCS score on arrival, high AIS score, and multiple traumas, all of which indicate a severe case, were almost the same between the groups.

**Table 1:** Information on patient background between meeting days and non-meeting days.

	Total	Non meeting days	Meeting days	p-value
	N = 7320	N = 5139	N = 2181	
**Age**				
Median (IQR), year	66 (43–78)	66 (44–78)	65 (41–77)	0.062
0–17, n (%)	543 (7.4%)	386 (7.5%)	157 (7.2%)	0.19
18–64, n (%)	2947 (40.3%)	2034 (39.6%)	913 (41.9%)	
65–, n (%)	3830 (52.3%)	2719 (52.9%)	1111 (50.9%)	
**Sex**				
Male, n (%)	4930 (67.3%)	3453 (67.2%)	1477 (67.7%)	0.66
**Type of trauma**				
Blunt, n (%)	7144 (97.6%)	5019 (97.7%)	2125 (97.4%)	0.55
**Mechanism of trauma**				
Traffic accident, n (%)	3214 (43.9%)	2230 (43.4%)	984 (45.1%)	0.39
Fall, n (%)	3480 (47.5%)	2467 (48.0%)	1013 (46.4%)	
Others, n (%)	626 (8.6%)	442 (8.6%)	184 (8.4%)	
**Transfer system**				
Ambulance, n (%)	6026 (82.3%)	4245 (82.6%)	1781 (81.7%)	0.47
Car/helicopter with physician, n (%)	873 (11.9%)	609 (11.9%)	264 (12.1%)	
Others, n (%)	421 (5.8%)	285 (5.5%)	136 (6.2%)	
**GCS at arrival**				
Median (IQR)	13 (8–15)	13 (8–15)	14 (8–15)	0.13
Mild, n (%)	3618 (49.4%)	2520 (49.0%)	1098 (50.3%)	0.57
Moderate, n (%)	1734 (23.7%)	1223 (23.8%)	511 (23.4%)	
Severe, n (%)	1968 (26.9%)	1396 (27.2%)	572 (26.2%)	
**Systolic BP on arrival**				
Median (IQR), mmHg	141 (120–164)	142 (120–164)	141 (120–164)	0.9
Hypotension, n (%)	479 (6.5%)	344 (6.7%)	135 (6.2%)	0.43
**Type of TBI**				
Focal brain injury, n (%)	201 (2.7%)	147 (2.9%)	54 (2.5%)	0.36
Diffuse brain injury, n (%)	2670 (36.5%)	1875 (36.5%)	795 (36.5%)	
Other, n (%)	4128 (56.4%)	2880 (56.0%)	1248 (57.2%)	
**Max head AIS**				
Median (IQR)	522 (7.1%)	384 (7.5%)	138 (6.3%)	0.2
3, n (%)	2468 (33.7%)	1727 (33.6%)	741 (34.0%)	0.083
4, n (%)	2998 (41.0%)	2074 (40.4%)	924 (42.4%)	
5, n (%)	1854 (25.3%)	1338 (26.0%)	516 (23.7%)	
**Multiple trauma**				
N (%)	2244 (30.7%)	1555 (30.3%)	689 (31.6%)	0.26
**ISS**				
Median (IQR)	20 (16–26)	20 (16–26)	20 (16–26)	0.57
**Operation for TBI**				
N (%)	1284 (17.5%)	913 (17.8%)	371 (17.0%)	0.44

*TBI* traumatic brain injury, *GCS* glasgow coma scale, *BP* blood pressure, *AIS* abbreviated injury scale, *ISS* injury severity score, *IQR* interquartile range.

### Mortality during meeting and non-meeting periods

Table 2 shows the proportion of in-hospital mortality and death in the ER, time from onset to arrival at the hospital, time from arrival to operation commencement, and duration of hospital stay during the meeting and non-meeting days. The proportion of participants experiencing in-hospital and ER mortality after TBI did not differ markedly between the non-meeting and meeting days (15.7% [808/5139] versus 14.5% [316/2181] for in-hospital mortality [adjusted OR, 0.93; 95% CI, 0.78–1.11], and 1.3% [66/5139] versus 1.4% [31/2, 181] for ER mortality [adjusted OR, 1.16; 95% CI, 0.73–1.84]). The time from onset to arrival at the hospital, time from arrival to operation commencement, and duration of hospital stay were similar between the groups (44 [33–63] vs. 43 [32–62] min, p = 0.28; 207 [106–4279] vs. 218 [106.5–4546.5] min, p = 0.69; and 14^5–34^ vs. 14^5–31^ days, p = 0.066; respectively).

**Table 2:** Outcomes between meeting and non-meeting days.

	Total	Non meeting days	Meeting days	p-value	Crude OR	95% CI	Adjusted OR	95% CI
	N = 7320	N = 5139	N = 2181					
Death at hospital discharge, n (%)	1124 (15.4%)	808 (15.7%)	316 (14.5%)	0.18	0.91	(0.79–1.05)	0.93	(0.78–1.11)
Death at emergency room, n (%)	97 ( 1.3%)	66 ( 1.3%)	31 (1.4%)	0.64	1.11	(0.72–1.70)	1.16	(0.73–1.84)
Time from onset to arrival at hospital, min	44 (33–63)	44 (33–63)	43 (32–62)	0.28				
Time from arrival to operation, min	210 (106–4361)	207 (106–4279)	218 (106.5–4546.5)	0.69				
Hospital stay days, day	14 (5–33)	14 (5–34)	14 (5–31)	0.066				

*OR* odds ratio, *CI* confidence interval.

Table 3 shows the TBI outcomes during the meeting and non-meeting days as per the type of national meeting. Even after adjusting for potential confounding factors, there were no significant differences in mortality during the meeting and non-meeting days (16.0% [230/1435] versus 12.7% [80/632] for JAAM meetings [adjusted OR, 0.76; 95% CI, 0.53–1.08]; 15.7% [170/1084] versus 15.4% [86/560] for JSICM meetings [adjusted OR, 1.06; 95% CI, 0.75–1.48]; 17.2% [222/1294] versus 15.7% [112/715] for JNS meetings [adjusted OR, 0.84; 95% CI, 0.61–1.16]); 14.5% [110/760] versus 14.0% [57/408] for JAST meetings [adjusted OR, 0.77; 95% CI, 0.49–1.23]; and 13.2% [110/831] versus 12.9% [54/418] for JSNT meetings [adjusted OR, 1.23; 95% CI, 0.80–1.88]). However, mortality associated with attendance at JAAM only tended to decrease on meeting days compared to non-meeting days [crude OR, 0.76; 95% CI, 0.58–1.00].

**Table 3:** Primary Outcomes between meeting days and non-meeting days as per the type of national meeting.

Death at hospital discharge	Total	Non meeting days	Meeting days	Crude OR	95% CI	Adjusted OR	95% CI
The Japanese Association for Acute Medicine, n/N (%)	310/2067 (15.0%)	230/1435 (16.0%)	80/632 (12.7%)	0.76	(0.58–1.00)	0.76	(0.53–1.08)
The Japanese Society of Intensive Care Medicine, n/N (%)	256/1644 (15.6%)	170/1084 (15.7%)	86/560 (15.4%)	0.98	(0.74–1.29)	1.06	(0.75–1.48)
The Japan Neurosurgical Society, n/N (%)	334 /2009 (16.6%)	222/1294 (17.2%)	112 /715 (15.7%)	0.9	(0.70–1.15)	0.84	(0.61–1.16)
The Japanese Association for the Surgery of Trauma, n/N (%)	167/1168 (14.3%)	110/760 (14.5%)	57/408 (14.0%)	0.96	(0.68–1.36)	0.77	(0.49–1.23)
The Japan Society of Neurotraumatology, n/N (%)	164/1249 (13.1%)	110/831 (13.2%)	54/418 (12.9%)	0.97	(0.69–1.38)	1.23	(0.80–1.88)

*OR* odds ratio, *CI* confidence interval.

## Discussion

Contrary to our hypothesis, there were no significant differences in mortality among TBI patients admitted in JTDB-registered hospitals during the national academic meeting and non-meeting days. To the best of our knowledge, the present study is the first to examine the “national meeting effect” on mortality in TBI patients. These results presented unique findings and might help medical professionals attend such academic meetings and learn from them without being concerned regarding this effect.

The “weekend effect” refers to the phenomenon in which patients admitted during weekends may have more fatal outcomes than those admitted during the weekdays^7,8^. Poor performances in hospitals are considered a reason for this effect. In terms of instances requiring time-critical intervention for better outcomes (acute myocardial infarction, cardiac arrest, and ischemic stroke), poor outcomes during off-hours have been observed with fewer aggressive interventional procedures, less subspecialty care, more complications, increased medical errors, and varied staff composition^28–30^.

Regarding the “meeting effect” on trauma, it has been reported that adjusted mortality did not increase significantly for patients admitted to trauma centers with American College of Surgeons trauma verification Level 1 during the conference versus non-conference days^6^. Although our study focused on traumatic brain injury, our results are consistent with those from previous studies on the “meeting effect” and trauma mortality using the JTDB database^31^. In a report using the JTDB database, both in-hospital mortality and death in the ER were significantly lower during the day than at night among emergency trauma patients; nevertheless, weekdays/weekends were not associated with either endpoint^7^. The reason why weekend effects on trauma were not obvious could be explained by the possibility that patients could immediately access the operation room or resources that might otherwise be occupied during normal business hours^7^. Similarly, the decline in the volume of scheduled surgery during the meeting periods might have contributed to the unchanged mortality.

We further assessed time differences as per the “meeting effect.” In our country, not all institutions have in-house neurosurgeons or trauma surgeons^32^. Hence, it is unlikely that high-risk patients were directed to such hospitals, possibly resulting in a longer duration from onset to arrival at the appropriate hospital or hospital arrival to operation commencement. However, we did not find any difference. Contrary to the aforementioned hospitals, JTDB-registered hospitals are accountable for “24 h a day, 365 days a year” available resources for injured patients even during national meetings, coupled with a sophisticated prehospital system that preferentially directs patients to the correct facilities. Advance assessment of staff composition and rotation during meeting days may help against the conference effect. Compared to other countries, Japan has a smaller land area and better transportation. The on-call attending surgeons head toward the hospital, responding immediately to trauma calls and provide consistent quality of trauma care even during meetings, reflected in the unchanged time from the arrival to operation. Furthermore, no difference in time outcome also contributed to the unchanged mortality during meeting days.

In our study, which focused on TBIs, only mortality associated with JAAM attendance tended to decrease on meeting than on non-meeting days; however, unlike previous studies, no significant differences were observed^31^. Although the definitive reasons for this remain unclear, this finding could be attributed to the fact that more ER physicians belong to the JAAM compared to the other four societies. Strong leadership, teamwork, and technical skills are essential for team performance and patient care in initial trauma management^33,34^, and more experienced ER physicians or trauma surgeons would encourage their hospitals to lead the treatment of hospitalized patients during academic national meeting dates^9^.

Considering TBI treatment, factors associated with consistent, high-quality care include appropriate staffing, prompt triage and decision making, and establishing inter-facility consultations. Trauma centers are required to be fully staffed and resourced irrespective of meetings^34,35^. Based on our results, medical professionals involved in TBI treatments in Japan can attend national academic meetings because through these meetings they might learn regarding the latest findings on TBI cases. Importantly, owing to the new coronavirus disease (COVID-19) pandemic, organizers of these conferences may consider offering optional opportunities for Web conferences so that trauma centers or ICUs would maintain their care capacities while medical professionals would benefit from educational opportunities that academic meetings provide. Therefore, it is important to continue monitoring the “meeting effect” concerning the survival of TBI patients.

Our study had several limitations. First, we did not address the influence of the location of the hospital providing the data. For instance, it has been shown that TBI mortality rates are higher in rural areas of the United States^35^. Therefore, our results might be different if we considered information regarding the regionality of hospitals. Second, we did not obtain detailed information on medical staff attending national meetings and non-participants at the hospitals. Additionally, we focused on TBI occurrence during five representative national meetings to simplify our research. The logical next step in this field of research would be the examination of both regional and international meeting periods. Third, the study primarily included major critical care cases; therefore, the results cannot be extended to other institutions. Fourth, this was an observational study, and other unknown confounding factors possibly existed. Finally, the validity and integrity of the data, and ascertainment bias, were potential limitations of our study. However, uniform data collection based on the JTDB registering system and large sample sizes should minimize these potential sources of bias.

## Conclusions

In this population, there were no significant differences in outcomes, such as in-hospital death, ED mortality, duration from onset to arrival at the hospital, duration of hospital arrival to operation commencement, and duration of hospital stay after TBI, between the meeting and non-meeting days.
